# A program of family-centered care for adolescents in short-term stay groups of juvenile justice institutions

**DOI:** 10.1186/s13034-017-0203-2

**Published:** 2017-12-19

**Authors:** Inge Simons, Eva Mulder, René Breuk, Kees Mos, Henk Rigter, Lieke van Domburgh, Robert Vermeiren

**Affiliations:** 1 Department of Child and Adolescent Psychiatry, Curium-Leiden University Medical Center, Post Box 15, 2300 AA Leiden, The Netherlands; 2 Intermetzo-Pluryn, Post Box 53, 6500 AB Nijmegen, The Netherlands; 3 Youth Interventions Foundation, Post Box 37, 2300 AA Leiden, The Netherlands; 4 Department of Child and Adolescent Psychiatry, De Bascule-VUmc, Post Box 303, 1115 ZG Duivendrecht, The Netherlands

**Keywords:** Family-centered care, Delinquent adolescents, Youth detention centers, Parental participation

## Abstract

**Background:**

To provide successful treatment to detained adolescents, staff in juvenile justice institutions need to work in family-centered ways. As juvenile justice institutions struggled to involve parents in their child’s treatment, we developed a program for family-centered care.

**Methods:**

The program was developed in close collaboration with staff from the two juvenile justice institutions participating in the Dutch Academic Workplace Forensic Care for Youth. To achieve an attainable program, we chose a bottom-up approach in which ideas for family-centered care were detailed and discussed by workgroups consisting of group leaders, family therapists, psychologists, other staff, researchers, and a parent.

**Results:**

The family-centered care program distinguishes four categories of parental participation: (a) informing parents, (b) parents meeting their child, (c) parents meeting staff, and (d) parents taking part in the treatment program. Additionally, the family-centered care program includes the option to start family therapy during detention of the youths, to be continued after discharge from the juvenile justice institutions. Training and coaching of staff are core components of the family-centered care program.

**Conclusions:**

The combination of training and the identification of attainable ways for staff to promote parental involvement makes the family-centered care program valuable for practice. Because the program builds on suggestions from previous research and on the theoretical background of evidence-based family therapies, it has potential to improve care for detained adolescents and their parents. Further research is required to confirm if this assumption is correct.

## Background

Treating incarcerated adolescents effectively requires involving their parents [22]. When treating delinquent youth, both protective and risk factors within the family domain must be addressed. Protective family factors include parental support, positive family interactions, personal assets of family members, future orientation of family members, and the family’s support network [6, 15]. Risk factors include lack of parental monitoring or inept discipline, poor family functioning, maltreatment, low family affection and warmth, and parental problems such as drug (ab)use, psychopathology, and criminal activity [6, 21, 33, 47]. If the family of the delinquent adolescent is not given appropriate attention, poor family functioning is likely to persist, influencing the prospect of the youth to get involved in the juvenile justice system [8, 9, 20, 34].

Involving parents in juvenile justice is considered important for promoting positive child and family outcomes [7, 53]. Family-centered approaches were shown to decrease youth recidivism [13, 24]. A recent meta-analysis has shown that adolescents with severe behavior problems benefit more from family therapy compared to their peers with less severe behavior problems [49]. Notwithstanding the evidence, there is a lack of active and positive parental involvement in the juvenile justice system [35]. Intervention programs offered to adolescents in youth detention institutions all too often do not adequately address the youth’s family [47]. Treatment instructions for involving parents of youths involved in the juvenile justice system are missing [7, 14, 29]. Until recently in the Netherlands, parents were kept at a distance and were hardly involved in their child’s treatment during detention in a Juvenile Justice Institution (JJI) [39, 50]. The resulting gap between home and the JJI is likely to impair rehabilitation after detention. When families are not engaged in treatment during detention, it is difficult to convince them to take part in family-based outpatient treatment interventions [32].

Realizing the importance of involving parents, Dutch JJIs incorporated a few family-oriented activities in their usual care program. These activities included staff calling parents once a week or inviting parents to key meetings where the intervention plan for their child is being discussed [46]. Although promising, JJIs were found to not properly adhere to these instructions for involving parents [18]. Ways to involve parents were not systematically implemented in practice and staff were not properly trained in working with parents. Therefore, in 2011, the Netherlands Government issued a national position paper encouraging JJIs to improve parental participation [39]. This paper however only sketched a broad perspective, which needed to be detailed for implementation in everyday practice. Therefore, we took up the challenge to improve care in JJIs by developing the program for family-centered care (FC). Most youths in JJIs are initially detained in a short-term stay group, for a maximum period of 90 days, awaiting the final ruling of the juvenile judge. The judge may decide that the adolescent is to be released, or to be detained longer. In the latter instance, the adolescent usually is transferred to a long-term stay group for detention lasting many months or years [40]. We developed two versions of FC, one for short-term stay groups and one for long-term stay groups. The present paper discusses the short-term stay version.

## Methods

The development of the FC program was one of the projects of the Academic Workplace Forensic Care for Youth (in Dutch: AWFZJ). The AWFZJ aims to bridge the gap between practice, research, education, and policy in forensic youth care by carrying out practice-based research. Two JJIs, two universities, two centers for child and adolescent psychiatry, and two universities of applied sciences in the Netherlands collaborate in this workplace to improve care for forensic youth and to reduce recidivism. The AWFZJ aims to translate research results into practice. In our study protocol paper, we describe the full background and methods of our study on FC [40].

We have developed the FC program in close collaboration with staff from the two JJIs participating in the AWFZJ. The family work in our program was based on the theory and practice of two evidence-based therapies, i.e., multidimensional family therapy, MDFT [26] and functional family therapy, FFT [2]. Main points of the underlying theory are [25, 37, 44]:

- The problem behavior of the adolescent, delinquency in this instance, is shaped by risk and protective factors from all major social domains of which he or she is part: the person himself, family, friends and peers, school and work, leisure time environments, and justice and probation authorities, including the JJI staff. These domains influence each other constantly and all these domains must be targeted to achieve lasting treatment success. Reinforcing protective factors will serve as a buffer against the influence of risk factors.
- Most adolescent problem behavior consists of a combination of troubles, e.g., delinquency, substance abuse, truancy, and comorbid mental health problems. Epidemiological models have shown that these problem behaviors tend to reinforce each other, which jeopardizes treatment attempts. Therefore, JJI staff and therapists need to address the full array of problems, at the individual level of the adolescent, and any other level, including the family.
- Family therapy has a relational focus. Besides focusing on the family and family relationships, the therapist also works with the other social domains. According to theoretical notions, lack of knowledge about problem behavior among youths, parents, and staff, family malfunctioning, and poor communication between family members all have been found to contribute to the incidence and persistence of adolescent problem behavior. This calls for (psycho-) education, training family members to properly communicate with each other, and training the parents in parental skills, such as setting and enforcing home rules.
- Key to effective interventions is motivating the adolescent and the parents to take part in FC and eventually in family therapy. Treatment motivation cannot be taken for granted. Motivating the adolescent and parents to join FC activities and family interventions takes time and requires a thorough understanding of the pathways leading to problematic behavior. The theory underlying family therapy further encourages the therapist to bond with both the adolescent and his parents in a committed, but neutral way. In other words, therapists—but also any other JJI staff—need to establish non-conflicting therapeutic alliances with both the youth and the parents.

**Fig. 1:**
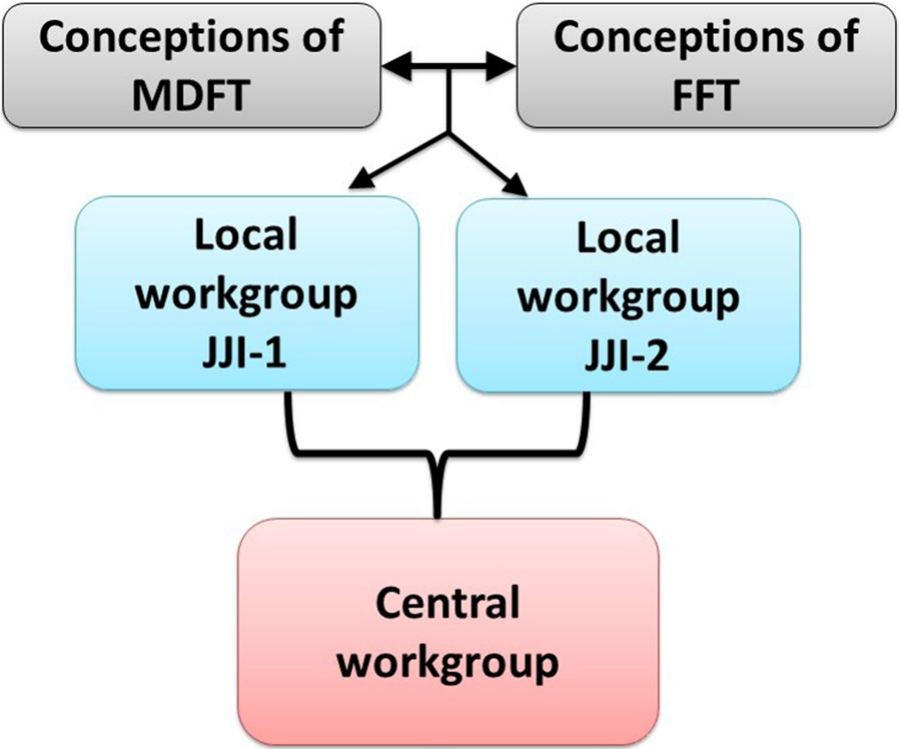
Bottom-up approach in devising the FC program

We discussed the family therapy insights in workgroups of JJI group leaders, family therapists, psychologists, other JJI staff, and researchers. Based on these insights, ideas for FC were detailed and discussed. As applicability in practice was an important goal for the AWFZJ, we chose a bottom-up approach for developing the FC program. Each of the participating JJIs had a local workgroup, of which representatives took part in a central workgroup (see Fig. 1). One parent attended the meetings of the central workgroup as an advisory member on behalf of the Dutch parents association for children with developmental disorders and educational or behavioral problems. In the workgroups, we strived to translate the theoretical background of family therapy [37, 44] and the broad perspective from the national position paper [39] into practice by providing guidelines and directions for family-centered care. The FC program is compatible with the usual care programs in JJIs in which only a few family-oriented activities were already incorporated [46]. The workgroups also developed training workshops for JJI staff.

## Results

The bottom-up workgroup sessions resulted in a manual describing how to deliver family-centered care in short-term stay groups in JJIs [31]. The manual starts by explaining the meaning of family-centered care: i.e., JJI staff actively involve parents in the guidance and treatment of their detained child. FC expects the entire institution to propagate family-centered care and all employees to embrace a systemic vision. In FC, staff work in a family-centered way. This starts as soon as the youth enters the JJI and continues throughout the stay. FC is integrated in all methods and procedures in the JJI and is therefore not considered to be a new form of therapy. Rather, FC changes practices for JJI staff regarding all youths and their parents. Therefore, FC is considered to be part of the basic program for delivering care in JJIs. Interventions within FC are selected according to the needs of adolescents and their parents. In FC, staff help families towards a better functioning. FC emphasizes that treatment gains during detention need to be maintained when the child returns home and recognizes that relapses are opportunities for change and growth. Therefore, staff help the adolescent to rehabilitate after discharge. Overall in FC, the trajectory during the youth’s detention is transparent to the adolescents and his parents, and staff understand the complexity of family-centered care in a closed facility. Because of the high variation in duration of adolescents’ stays, FC does not follow fixed time schedules; the activities are scheduled according to the needs of the adolescent and his parents during detention. FC offers much room for tailoring by group workers.

FC aims to improve parental participation rates, first by training staff in family-centered work according to the theoretical principles outlined above. The purpose of the training is for staff to increase systemic competencies and to develop a systemic perspective, i.e., being constantly aware of the importance and relevance of social domains, most notably the family, to prevent the youth from relapsing into problem behavior. In the systemic perspective, adolescents are seen as part of a family and this family is part of the solution for the current crisis.

Implementing FC introduces a different approach of treating detained adolescents. Involving parents in their child’s everyday life and throughout their child’s detention becomes routine in JJI procedures. This involvement is operationalized by the following activities: (a) informing parents; (b) parents meeting their child; (c) parents meeting staff; (d) parents taking part in the treatment program. Each activity will be explained in detail below. Through involving parents in every aspect of their child’s detention, FC aims to increase youths’ and parents’ motivation for treatment interventions. Theories underlying family therapy see reconnection of the parents and child as a strong boost for treatment motivation. The four sets of activities in FC serve to reconnect the family members, and are therefore considered crucial for achieving positive treatment outcomes. If involving parents is routine and if staff establish working alliances with youths and parents, youth may be more willing to accept their parents’ participation, both may feel more appreciated, and parents may be more motivated for participation.

### Family-centered care: informing parents

In FC, parents are provided with adequate and timely information on procedures, developments, and events. Parents are contacted by telephone on the first day their child enters the JJI. The person best suited for making this call is the mentor; the group worker who has been assigned to the adolescent concerned. In this first contact, the mentor stresses that the best way to effectively treat the adolescent, is with the help of the parents. The mentor explains the importance of parents’ involvement during their child’s stay in the JJI. From there on, the mentor has at least weekly telephone contact with the parents to ensure that they monitor their child’s behavior in the JJI and the progress made in achieving the treatment goals.

In addition to the calls by the mentor, the child’s psychologist, or pedagogue (hereafter jointly referred to as psychologist), informs the parents about the nature of their child’s problems, and about psycho-education and treatment opportunities.

### Family-centered care: parents meeting their children

One goal of FC is to increase parents’ motivation to visit their child frequently. By Dutch law, parents have a privileged status in visiting their children in a JJI. In FC, the opportunities for parents to visit their child are no longer restricted to the regular visiting hours, as parents are actively invited to engage in their child’s everyday life in detention. Parental participation moves beyond seeing the youth in the visiting room. Parents are offered a tour through the JJI and are invited to attend activities of the so-called "living group" in which their child has been placed. Some of these activities that are open to parents are organized on a regular basis, such as family evenings. Other group-based activities are more spontaneous and less structured, tailored towards the needs of the youth and his parents. Examples of the latter are cooking and/or dining, game nights, or celebrations of birthdays or of diplomas obtained. Parents are encouraged to play a part in their child’s everyday life in the JJI in the hope that the family bond will strengthen and communication will improve, through which trust can rebuild. This provides families with the opportunity to share positive experiences.

### Family-centered care: parents meeting the staff

In the first week of detention, the mentor calls the parents and schedules a so-called family meeting for the third week, to be attended by the parents, the youth, the mentor, and the psychologist. If, based on the available information about the family, the meeting is expected to be complicated, the psychologist may consult a family therapist in advance. If needed, the latter is available to assist during the family meeting.

At the beginning of the family meeting, the psychologist first sits down with the parents alone to welcome them and to make them feel at ease. The psychologist stresses how important parents are for their child, and for the JJI to provide the best care and treatment. Spending time with the parents enables the psychologist to learn about the family history, and about family-based protective and risk factors, and other important domains shaping the adolescent’s behavior. After half an hour, the mentor and the adolescent join the meeting. The second part of the family meeting allows the parent and child to interact with each other in a positive way (to be encouraged by the psychologist and the mentor). At the same time, it allows the psychologist to observe the family dynamics. This information will later be used in the treatment. A third part of the meeting serves to discuss the adolescent’s problem behavior and the content of the treatment plan to be drafted. Shared-decision making is encouraged; input in this plan from the parents and the adolescent is required and essential for increasing treatment motivation. For as long as the adolescent stays in the JJI, the parents are invited to follow-up meetings with the psychologist, the mentor, and the adolescent to evaluate the progress according to this treatment plan.

### Family-centered care: parents taking part in the treatment program

In FC, parents are always informed about their child’s treatment program. Along the course of the adolescents’ treatment, parents are invited to participate in their son’s therapy sessions. Intervention programs such as aggression regulation training, social skills training, and offense analysis, often have their own terminology. To ensure that parents are able to communicate with their child about the therapy, parents join special sessions to learn the so-called “intervention language”. Additionally, during the child’s stay, staff pay attention to family relationships, communication, and dynamics, coaching both the adolescent and his parents towards more positive interactions.

**Fig. 2:**
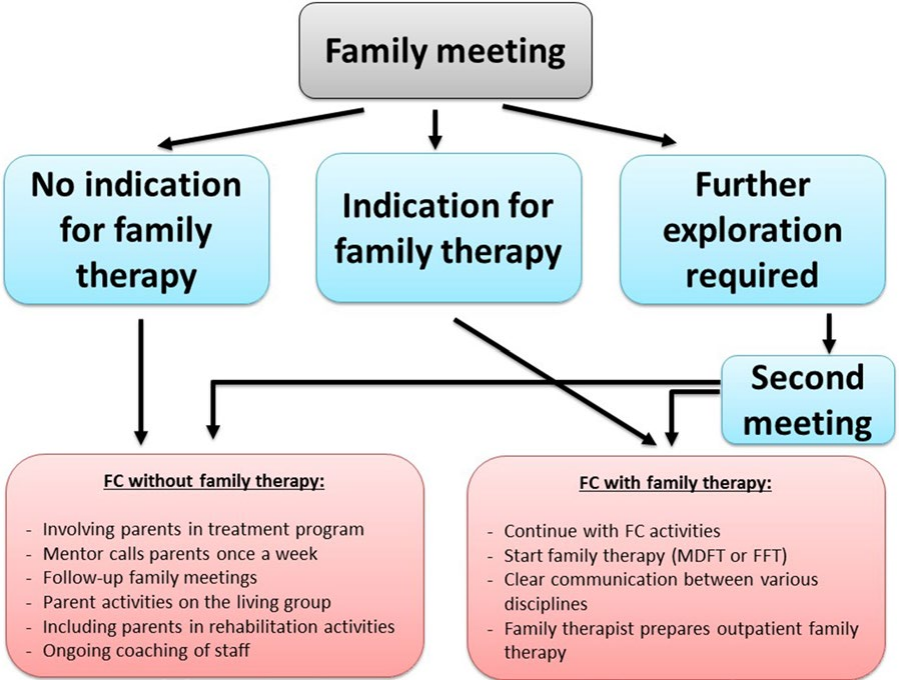
Routes in FC on short-term stay groups

In the first family meeting, JJI staff pay attention to the risk and protective factors influencing the problem behavior of the youth. Based on their findings, three trajectories are possible, see Fig. 2.

1. FC without family therapy.
2. In FC, family therapy starts during detention and continues after discharge.
3. Further exploration is required to decide upon the appropriate trajectory.

If family therapy is not indicated (first route), staff involve parents according to the above-described principles of FC and invite parents for family activities as described in the program manual.

In the second route, family therapy (FFT or MDFT) starts as soon as possible and continues as outpatient therapy when the adolescent is discharged from the JJI. The type of family therapy to be chosen does not depend on theoretical considerations, but on the availability of either therapy within the JJI concerned. We assured that our FC program fits to both forms of family therapy. For the first residential phase, family therapy is adapted for use in closed settings such as JJIs [32]. The family therapist schedules frequent family sessions and individual sessions with the youth or the parents. Within FC, family therapists adhere to the MDFT or FFT manual, while there is some degree of flexibility regarding the frequency of sessions depending on the needs of adolescents and their parents. During detention, family therapy aims to improve the relationship and communication between the family members. When the youth returns home, real-life practice for improving family functioning begins.

In case further exploration of the family process is required as in the third route, a second meeting is scheduled on short notice to thoroughly assess the topics at hand. This route is applicable in three circumstances. In first instance, important family themes need to be discussed before juvenile discharge, e.g., crises within the family or questions about living arrangements other than with parents. In the second case, the psychologist has doubts about whether family therapy is indicated and needs another meeting to make an informed decision. In last instance, family therapy is indicated but extra sessions are required to boost the family members’ motivation to engage in family therapy. In all circumstances, the psychologist consults with the family therapist who is available to assist during or preparing for the second meeting.

### Training staff in FC

The one-day training aims to familiarize staff with the principles of FC, to increase systemic competencies, and to ameliorate the implementation of family-centered work according to the FC manual. The training empowers staff to motivate parents for involvement. Once parents are engaged, bridges are built between family members and staff; between home and the JJI. During the training, special attention is paid to equip mentors of adolescents to motivate parents to visit their child in the JJI, as a mentor is the primary contact person for parents. Mentors are trained to contact, inform, and involve the parents. The training helps staff to adopt a systemic perspective and basic conceptions of family systems theory are explained. In the training, staff learn to see parents as supportive persons who do their best to deal with a difficult situation, and who are essential for establishing positive treatment outcomes. Staff learn about the two-way interaction patterns between parents and their children and how to build multiple therapeutic alliances, i.e., having a good bond with the youth and the parents alike, without taking sides.

Through role-playing exercises, group workers and psychologists train their skills in communicating with families, in person and through telephone contact. Additionally, family meetings are practiced through which staff experience how to establish multiple therapeutic alliances. The training provides staff with tools in reframing, improving the interrelationships between family members, increasing hope and motivation for change, and reducing negativity and blaming while improving positive communication between family members. Psychologists receive a specialized one-day workshop to enhance their skills required for the family-focused assessment during the family meeting.

The training program for staff includes bi-annual booster sessions to ensure that skills are practiced, improved, and fine-tuned. These booster sessions take up halve a day in which trainers repeat information from the original training and evaluate the current state of affairs regarding family-centered work in the teams. Teams of staff members reflect on which aspects of FC go well, and on which aspects need improvement. The trainers use this information to shape the training into a customized program tailored to the needs of a specific team.

Besides the training and booster sessions, FC prescribes team coaching supervised by a family therapist. This coaching takes place during the team meetings, which are scheduled every other week in the JJI. The first team meeting reserves one hour for so-called “intervision”. During this intervision, group workers each present a problem or question regarding contact with parents on which he or she would like to receive feedback. One of the cases is selected for an in-depth discussion with colleagues, promoting systemic competencies and family-proof solutions for the problem. The other team meeting reserves one hour for discussing the case from a systemic perspective; attentive to the family the youth originated from and, in most instances, will return to.

## Discussion

We succeeded in developing a program of family-centered care (FC) for adolescents in short-term stay groups of JJIs [31]. Our FC program changes the way in which parents are involved during their child’s detention. The program moves beyond basic visitations for parents in the impersonal visiting room, towards parents being part of their child’s everyday life in the JJI. In FC, parents are actively invited to play a prominent role during their child’s detention and in their treatment. This involves being informed of every intervention, being part of decisions to be made, visiting the adolescent in his living group, taking part in living group activities, and joining meetings for parents. In addition, the FC program offers the opportunity to start family therapy during detention and to continue it on an outpatient basis after detention. Overall, training in FC changes the way in which JJI staff think about parents, which will be reflected in their work. The FC program is not only of interest for JJIs, but is easily translated to other residential settings as well. For example, the program has recently been adjusted for residential care institutions [41].

We expect FC to be successful because of its evidence-based background in which the program meets suggestions from previous studies. First and foremost, the FC program stimulates parental involvement, as is advocated by several previous researchers [1, 5, 13, 16, 52]. Other researchers stated that children should be seen as belonging to the families and that contact between children and family members should be considered as a right, not as a privilege [12, 36]. Residential care should persevere and, if possible, strengthen the connections between children and their family members [43]. Our FC program incorporated these views. Enabling parents to spend time with their child in the JJI provides families with the opportunity for positive experiences and to engage in positive communication, which in turn strengthens the family bond. This helps rebuilding trust and hope for the future [27]. Second, the FC program emphasizes the importance of telephone contact with parents initiated by JJI staff on the first day of the child’s detention. This first contact is the beginning of building a relationship between staff and parents and sets the stage for successful parental involvement [19]. Third, the family meeting enables staff to learn about parenting practices, family process, peer influence, and adolescent-specific characteristics [42]. As parents usually are the most reliable source of information about their children [13, 38], this meeting results in a better insight in the adolescent’s problems. The family meeting might have an immediate therapeutic effect as well. If adolescents see how their offending behavior hurts family members, it is likely to increase their motivation for behavioral change and to promote a positive focus on the future [30]. Fourth, the FC program encourages shared decision-making, which has previously been identified as part of the central focus of family-centered care [43]. Fifth, the FC program emphasizes the importance of tailoring interventions to the risk and protective factors within the family and to the needs of the adolescent and his family, as suggested by previous research [23]. Sixth, the FC program offers the opportunity to start family therapy during detention which can continue on an outpatient basis, as is also previously advocated by other researchers [1, 48]. Finally, the program is part of a package deal including training of staff. One of the building blocks of implementing FC in practice is increasing systemic competencies among staff [4]. In FC training, staff learn about the mutual influence between youth problem behavior and family functioning, learn to see the family as part of the solution for the current crisis, and to build therapeutic alliances with parents. These themes and tools in the training are in line with recommendations for family-centered work [3, 10, 12, 14, 17, 28, 32, 50, 51], which might result in staff who are more sensitive in working with parents [45]. The training includes role-play exercises, enabling staff to train their skills in working with families, both in person and through telephone contact [19].

Before the start of our project, JJIs in the Netherlands reached unsatisfactory levels of parental participation [18, 39, 40, 50]. Bearing this in mind, we realized that our FC program did not only need to be strongly evidence-based, but also had to be attentive to the attainability of our program in practice. Our bottom-up approach contributed to achieving our aim, although this is not enough to reach successful implementation in practice. In order to truly work in a family-centered way, JJIs need to fully embrace a family-centered approach. Successful implementation is only possible if all layers and disciplines of the institution adopt a systemic view and develop skills in working with families [32]. Previous research has emphasized that the implementation of new interventions is challenging, especially in the case of family-focused interventions for youth with behavioral problems [5, 45]. Therefore, JJIs are encouraged to follow our bottom-up strategies to motivate staff for FC and to take the time to train staff in FC. The entire organization needs to be prepared for the implementation of a new program [11]. Overall, if implemented carefully, the FC program has great potential for improving care for detained adolescents and their families. Improved care through FC might contribute to positive treatment outcomes and FC ensures a better connection with outpatient care after detention. Careful and successful implementation is a requirement for FC to live up to its potential. Whether FC is able to improve care for detained adolescents and their families, will be examined in a practice-based mixed methods study [40]. In this study, we will address the following hypotheses comparing FC with usual care during detention: (1) FC increases parents’ involvement with their detained child; (2) FC increases the motivation of the adolescent and his parents for accepting treatment and guidance by JJI staff and for taking part in family meetings; (3) FC adolescents show less problem behavior; (4) FC improves family interactions; (5) FC parents experience less parenting stress; (6) FC youths more often return to their family’s home upon discharge; (7) FC enhances adolescents’ and parents’ satisfaction with the JJI; and (8) in FC groups, JJI staff members are more satisfied, feel more confident in their contact with parents, and more often incorporate the family perspective in their thinking [40].

## Abbreviations

AWFZJ: Academic Workplace Forensic Care for Youth
FC: family-centered care
FFT: functional family therapy
JJI: Juvenile Justice Institution
MDFT: multidimensional family therapy
